# Testosterone-Sparing Treatment Strategies for Biochemically Recurrent Prostate Cancer After Radical Prostatectomy

**DOI:** 10.1007/s11912-026-01776-5

**Published:** 2026-03-31

**Authors:** Faris Najdawi, Rashid K. Sayyid, Thomas Ahlering, David I. Lee, Ryan W. Dobbs, Mohammed Shahait

**Affiliations:** 1 Division of Urology, Cook County Health and Hospitals System, Chicago, IL USA; 2 Department of Urology, University of Arizona, Tucson, AZ USA; 3 Department of Urology, University of California at Irvine, Irvine, CA USA

**Keywords:** Prostate cancer, Biochemical recurrence, Testosterone-sparing treatment, Androgen deprivation therapy

## Abstract

**Purpose of Review:**

Biochemical recurrence (BCR) after radical prostatectomy occurs in up to one-third of patients and increases the risk of metastasis and prostate cancer–specific mortality. While androgen deprivation therapy (ADT) remains a foundational approach, its routine use in low- and intermediate-risk BCR raises concerns regarding overtreatment and long-term quality-of-life (QOL) impairment. This review summarizes contemporary testosterone-sparing treatment (TST) strategies for BCR, including patient selection frameworks, emerging therapeutics, and evolving imaging modalities.

**Recent Findings:**

Advances in multifactorial risk stratification—including genomic classifiers and artificial intelligence (AI)-driven models—are reshaping treatment decision-making and may help identify patients unlikely to benefit from ADT. PSMA-PET has redefined disease staging and is increasingly used to guide salvage radiation therapy and metastasis-directed therapy. Several emerging TST strategies—including enzalutamide monotherapy, salvage radiation without ADT, metastasis-directed therapy, salvage lymph node dissection, radioligand therapy, and structured lifestyle interventions—demonstrate potential to delay or avoid hormonal therapy. Early evidence suggests select patients can maintain cancer control while avoiding testosterone suppression and its associated toxicities.

**Summary:**

BCR represents a heterogeneous clinical state, requiring individualized management strategies that balance oncologic efficacy with long-term survivorship concerns. Testosterone-sparing approaches may provide safe alternatives to ADT in appropriately selected patients. Ongoing work to validate predictive biomarkers and integrate AI-derived decision tools is expected to refine patient selection as emerging clinical trials mature.

## Introduction

Radical prostatectomy (RP) remains a guideline-recommended treatment option for the treatment of appropriately selected patients with clinically localized prostate cancer. However, even with careful patient selection, biochemical recurrence (BCR) occurs in approximately 35% of patients within 10 years of primary treatment, with even higher rates reported for patients with high-risk clinicopathologic features [1–3]. BCR is associated with increased risks of metastatic disease progression (MDP) and prostate cancer-specific mortality (PCSM) [4]. It is defined as a post-operative prostate-specific antigen (PSA) level of *≥* 0.2 ng/mL. The traditional management approach for BCR includes an extended course of androgen deprivation therapy (ADT) and post-operative radiation. This paradigm has shifted with current literature, which has been reflected in the most recent 2024 American Urologic Association (AUA)/American Society for Radiation Oncology (ASTRO)/Society of Urologic Oncology (SUO) Guideline on Salvage Therapy for Prostate Cancer, which notes that clinicians may offer radiation therapy (RT) without ADT for patients with BCR after RP without any high-risk features [5]. This guideline also highlights the impact of ADT on patients’ quality of life (QOL), advising that clinicians should discuss ADT treatment adverse effects including decreased libido, erectile dysfunction, hot flashes, and fatigue as well as the impact of medical comorbidities associated with treatment [5]. The chronic side effects of ADT include anemia, osteoporosis, fractures (relative risk [RR] 1.45), obesity, sarcopenia, lipid alterations, insulin resistance and diabetes (hazard ratio [HR] 1.44). ADT is also associated with other adverse effects, including cardiovascular disease (HR 1.20), thromboembolism, colorectal cancer, depression, dementia (HR 1.17), and renal failure. The incidence of these adverse effects increases with longer-term ADT use [6–10].

Despite the established role of ADT in the management of high-risk and advanced prostate cancer, it is frequently overused, particularly in patients with BCR or low-risk disease, despite limited evidence of clinical benefit in these settings [11]. Overtreatment exposes patients to significant toxicity and potential side effects. De-implementation strategies are essential to reduce ADT use where it offers no meaningful clinical benefit, while prioritizing cancer control and patient safety.

The adverse effects of ADT significantly impact patient QOL and raise concerns of overtreatment [12]. Specific reported rates of ADT discontinuation due to adverse effects in the general population are lacking. But in clinical trials in which an ADT control group was used, discontinuation rates due to adverse effects have been reported in 9-27.9% of patients [13, 14]. This has led to the adoption of testosterone-sparing treatment (TST) strategies as an alternative approach to ADT in select BCR cases. Advances in imaging, diagnostic testing, and treatment modalities, in addition to a growing body of evidence, including epidemiological studies and retrospective reviews, have shown that while ADT improves overall survival outcomes, not all patients experience the same benefit from additional treatment. As BCR disease is heterogeneous, and response to ADT is variable among patients, these tools may be helpful in risk-stratification and to identify patients who may benefit from TST approaches. The purpose of this review is to describe the current literature supporting TST strategies for BCR after RP.

## Biochemical Recurrence: Classification and Risk Stratification

### Tools Based on Clinicopathological Variables

Multiple risk-stratification systems and prognostic models have been proposed to risk-stratify BCR patients (high versus low risk) and to help guide a risk-stratified treatment approach. These include the Cancer of the Prostate Risk Assessment Score (CAPRA-S), Memorial Sloan Kettering Post-Treatment Nomogram, and European Association of Urology (EAU) BCR risk grouping, which are reliant on key features, including [15–17]. High-risk patient and disease characteristics include short prostate-specific antigen doubling time (PSADT), presence/absence of node-positive disease, short interval from RP to BCR, grade group (GG), and pathologic stage. These risk stratification tools are summarized in Table 1.

**Table 1:** Prostate cancer BCR risk assessment tools

Tool	The Cancer of the Prostate Risk Assessment Score (CAPRA-S) [16]	Memorial Sloan Kettering Post-Treatment Nomogram [15]	EAU BCR Risk Stratification Tool [17]
Type	Scoring System	Predictive Nomogram	Risk Group Classification
Variables	PSA Pathologic GS Surgical margin SVI ECE LNI	Pre-op PSA Pathological GS ECE SVI Surgical margin LNI Time to BCR Age	PSADT Pathological GS Time to BCR
Scoring / Risk Groups	LR: 0–2 points IR: 3–5 points HR: 6–12 points	Continuous probability score (0–100%)	LR = GS < 8 or ISUP < 3, and PSADT > 12 months HR = fails to meet LR criteria
5-year PFS	5-year PFS for: LR = 72% IR = 39% HR = 17%	Variable	*LR*: 5-year PCSS = 99.7% 5-year MFS = 97.5% *HR:* 5-year PCSS = 93.8% 5-year MFS = 86.7%
Validity	5-year PFS c-index = 0.73 PCSM c-index = 0.85	5-year PFS AUC = 0.894	PCSM c-index = 0.69

Available risk assessment tools for biochemically recurrent prostate cancer

### Tools based on Emerging Tissue Biomarkers

Genomic classifiers have also shown promise in identifying more aggressive BCR disease. Three commercially available tests are Decipher, Oncotype DX, and Prolaris, with each showing promise in improving prognostic ability in the post-RP BCR setting [18]. Each test provides a score based on the expression of a panel of genes in a patient’s prostatectomy or biopsy specimen which can be used to predict prognosis. Decipher^→^ (Myriad Genetics, Salt Lake City UT) has proven to be reliable in predicting metastatic progression post-RP and improve prognostic performance of risk models [19, 20]. This genomic classifier has been incorporated into a risk-stratification model identifying patients who would most benefit from adjuvant radiation therapy (aRT) and has since been externally validated [21, 22].

A multimodal artificial intelligence (MMAI) deep-learning model has shown promise in identifying prognostically valuable biomarkers, improving risk stratification systems, and enhancing clinical outcome prediction [23–25]. The ArteraAI-created MMAI model (Artera, Los Altos CA) was developed using deep learning on pretreatment prostate tissue histopathology and clinical data from 5727 patients from five Phase 3 randomized trials to identify a biomarker which could identify patients who would benefit from the addition of ADT to RT. This model has been validated for patients with BCR after RP by using histopathology and clinical data from two landmark trials, NRG/RTOG 9601 and 0534, to estimate the risk of distant metastases (DM) and predict the benefit of adding ADT to RT [26]. It has been shown to be superior to the National Cancer Center Network (NCCN) risk stratification tool in predicting DM, BCR, PCSM, and OS [23]. Importantly, it classifies significantly more patients as low risk (LR) (43.5% of total patients compared to 30.4% based on NCCN risk), while maintaining comparable rates of DM at 10 years [25]. Moreover, it showed that while one-third of patients with intermediate risk BCR had improved outcomes with ADT, two thirds of patients did not [24]. Ultimately, this MMAI model may enhance patient counseling and QOL by identifying those who can safely avoid or shorten ADT, particularly in the controversial intermediate-risk group. Further work is needed to establish standardized risk cutoffs, and the model remains investigational for BCR disease.

However, it is important to note that the AUA/ASTRO/SUO guideline states the ability of a genomic score to inform the likelihood of response to salvage radiation therapy (sRT) is yet to be determined and, as such, does not recommend reflexive use of genomic testing for patients being considered for sRT [5]. Furthermore, it does not comment on genomic classifiers or MMAI models’ value in predicting response to aRT.

### Emerging Role of PSMA-PET Scan in BCR

The introduction of prostate-specific membrane antigen (PSMA)-PET scan has redefined the approach to treating BCR disease thanks to the improved sensitivity and specificity for disease detection, compared to conventional imaging (CI) [27, 28]. PSMA-PET has proven to be a prognostic biomarker marker of MDP; metastasis-free survival (MFS), which is three times shorter in men with PMSA-positive lesions at the time of BCR as compared to those without PSMA-positive lesions, with identical PSA levels at the time of BCR (*p* = 0.006; HR: 2.39) [29]. Due to its higher sensitivity and specificity, PSMA-PET allows for more comprehensive treatment planning for sRT. Several radiotracers have already been FDA-approved in the BCR disease setting [30–32]. But although PSMA-PET outperforms CI in diagnostic accuracy, its sensitivity remains relatively limited in the post-RP BCR setting, especially at lower PSAs < 0.5 ng/mL where detection rates ranged from 36 to 63% depending on the radioligand used [33–35]. Therefore, there is continued need to identify prostate cancer-specific cell targets for both diagnostic and therapeutic benefit.

One such radioligand currently under investigation utilizes copper isotopes for imaging (Cu^64^) and therapy (Cu^67^) and has two PSMA-targeting functional groups. Cu^64^ has a significantly longer half-life (12.7 h) than either gallium ^68^Ga (1.1 h) or piflufolastat (^18^F), and a shorter positron range (0.56 mm) [36]. In a phase I/II trial for BCR disease, ^64^Cu-SAR-bisPSMA was shown to be safe and effective, detecting lesions *≥* 2 mm in 80% of patients [36]. Studies evaluating the diagnostic accuracy of ^64^Cu for BCR disease are ongoing [37]. These advances in imaging will enable more precise and personalized TST approaches.

### Testosterone-Sparing Treatment Options

Several TST strategies for BCR exist including active surveillance (AS), sRT and aRT without concurrent ADT, second-generation nonsteroidal anti-androgen monotherapy, metastasis direct therapy, and targeted radioligand therapy (RLT).

### Active Surveillance

The natural history and progression of BCR after RP has been well documented with several large retrospective studies [1, 38–40]. It has been consistently demonstrated that BCR is a poor predictor of prostate cancer progression and not all BCR will lead to death [41]. In fact, in men with BCR, five-year PCSS is 90% with local recurrence only, 70% with nodal disease, and 47% with metastatic disease. Importantly, these results are with CI [42]. Additionally, adjuvant treatment will not affect PCSM in all patients and any treatment risks increasing morbidity and decreasing QOL for a subset of patients. As such, the aim is to identify patients with BCR who would potentially benefit from additional therapy and preserve QOL in those who will not. Advanced patient age, increased GS, advanced tumor stage, and shorter PSADT are all independent predictors of systemic progression and PCSM, with GS being the strongest predictor of both progression and PCSM [38]. These results support similar large population study findings, reflecting a relatively high PCSS among patients with favorable disease characteristics [43]. These findings should be taken into consideration when counseling patients on their treatment options, in addition to the adverse effects any treatment inherently entails. While we are waiting on more data to mature, the ideal patient for AS has low risk features, negative PSMA-PET scan, and low genomic classifier score (GCS).

### Metastasis-Directed Therapy

Local ablation for oligometastatic disease with metastasis-directed therapy (MDT) has proven curative across many disease sites including breast and colorectal cancer [44, 45]. Recent advances in imaging allows for earlier and more accurate identification of recurrent disease, providing the opportunity for earlier and more comprehensive treatment. Pooled data from the STOMP and ORIOLE trials showed MDT for BCR prostate cancer with stereotactic body radiotherapy (SBRT) or metastasectomy improves PFS when compared to AS (5.9 months versus 11.9 months; HR: 0.44, *p* < 0.001) [46]. Moreover, data from the ORIOLE trial demonstrated the importance of next generation imaging and treating all PMSA-avid lesions for maximum oncologic benefit, showing men with any untreated lesions had significantly worse six-month PFS [47]. Metastasis-directed RT to PSMA-PET-avid recurrent disease reduces the rate of subsequent DM and improves identification of patients who will benefit from treatment intensification [48, 49]. The implication of these findings is select patients with oligometastatic disease may be able to undergo targeted therapy with high accuracy, minimizing radiation toxicity, and possibly reducing the need for systemic therapy.

### Salvage Pelvic Lymph Node Dissection Without ADT

Separately, the benefit of salvage pelvic lymph node dissection (sPLND) has been evaluated in patients with lymph node-only recurrent disease [50–55]. The reported efficacy of sPLND has been variable, with two-year and five-year PFS rates ranging from 23 to 64% and 6–31%, respectively [54, 55]. In a single institution study utilizing robot-assisted PLND, 20% of patients sustained complete biochemical response (BR), defined as PSA < 0.02 ng/mL, at 15 months [50]. Several pre-operative predictors of early (< 12 months) clinical progression have been identified: grade group 5 disease (HR 2.04), retroperitoneal uptake on PET imaging (HR 1.24), *≥* 3 positive lymph nodes on PET/CT (HR 1.26), and PSA level at time of sPLND [54]. Importantly, the type of imaging used in determining the extent of disease spread has proven critical. Patients diagnosed with ^68^Ga-PSMA had a higher rate of BCR after sPLND compared to ^18^F (45.7% versus 21.7%; *p* = 0.040) [53].

Currently, sPLND represents a potential treatment option for pelvic lymph node-localized BCR disease. However, prospective randomized clinical trials using standardized next-generation imaging are needed to determine the long-term clinical outcomes and define its role. Furthermore, consideration as to when or if nodal dissection should be carried out to retroperitoneal nodes is needed and should be evaluated.

### Salvage and Adjuvant Radiation Therapy Without ADT

Adjuvant RT is given shortly after surgery in patients without evidence of BCR with the goal of preventing disease recurrence, while sRT is given after BCR has occurred. There is strong evidence to support the addition of ADT to sRT for BCR disease with HR clinicopathologic features, and its benefits on progression-free survival (PFS), metastasis-free survival (MFS), and OS [56–59]. But further subgroup analyses have shown limitations to the benefit of adding ADT to sRT. In RTOG 9601, sRT to the prostatic bed and membranous urethra with ADT was associated with improved OS, compared to no ADT, for GS > 8 (HR 0.76) and GS 7 (HR 0.76) disease, but there was no improvement in OS for patients with GS *≤* 6 (HR 0.95). On post hoc analyses, there was no OS benefit for patients with PSA < 0.7 ng/mL at the time of sRT (HR 1.13; *p* = 0.53), nor for those with negative surgical margins (HR 0.87; *p* = 0.56). Importantly, a secondary analysis of RTOG 9601 found the addition of ADT to sRT in patients with PSA < 0.7 ng/mL significantly increased other-cause mortality (HR 1.94; *p* = 0.01) and increased odds of cardiac and neuro-toxic effects [odds ratio (OR) 3.57; *p* = 0.05] [60]. In another secondary analysis, a genomic classifier (Decipher^→^) was used on samples from 352 patients. In early sRT patients, defined as PSA < 0.7 and low GCS < 0.45, adding ADT to sRT provided limited benefit to PCSM and OS (2.4% absolute improvement in 12-year OS compared with 8.9% improvement in those with high GCS) [61]. These findings are highlighted by the AUA/ASTRO/SUO guideline recommendation against the addition of ADT to sRT in patients without HR features [5].

The addition of targeted sRT to pelvic lymph nodes (PLNRT) has also proven beneficial when compared to prostatic bed sRT (PBRT) alone. PLNRT plus PBRT plus short-term (4–6 months) ADT resulted in a significantly improved five-year PFS of 87.4%, compared to both PBRT plus ADT (81.3%; HR 0.70) and PBRT alone (70.9%; HR 0.52) [58]. Moreover, the addition of PLNRT reduced the five-year risk of disease progression by 30% compared to PBRT plus ADT.

Importantly, the optimal use and duration of ADT alongside sRT remains uncertain in the absence of a validated predictive model. The advent of AI-based predictive models may help in answering these questions. ArteraAI’s MMAI model has already shown promise in identifying patient subsets who benefit from the addition of ADT to primary RT and in guiding ADT duration [24, 62]. Though it has yet to be validated in the BCR disease setting. Currently, a phase 3 trial is currently underway aiming to identify men with BCR disease post-RP who can safely reduce or avoid ADT (NCT06274047).

### Enzalutamide Monotherapy

The introduction of next-generation androgen receptor inhibitors (ARi) has provided another tool for the management of non-metastatic, high-risk BCR patients, highlighted by the landmark EMBARK trial [63]. Since its FDA approval in 2012, enzalutamide monotherapy has been shown to achieve a durable PSA response while maintaining testosterone levels and reducing the impact on patient QOL compared to ADT [64]. Moreover, adverse effects of ARi differ from traditional ADT, with ARi having a lower frequency of hot flashes and minimal impact on bone mineral density, contrary to ADT [63, 64].

### Targeted Radioligand Therapy

PSMA-targeted RLT offers a unique non-hormone treatment modality for BCR prostate cancer. Currently reserved for metastatic castration resistant prostate cancer, RLT has proven beneficial in improving OS and PFS [65, 66]. While literature on early RLT in BCR disease is lacking, a clinical trial (NCT04206319) is currently underway utilizing radium-223, an alpha-emitting radioligand that accumulates in areas of high bone turnover [67]. Patients with BCR receive RLT with radium-223 and without ADT. The study is expected to be completed in late 2025, with highly anticipated results.

^67^Cu-SAR-bisPSMA has also shown promise in RLT. It has yet to be studied as a therapeutic option for BCR disease, but it did receive Fast Track Designation by the Food and Drug Administration (FDA) in February 2025 for treatment of metastatic castration-resistant prostate cancer [68].

### Diet, Supplement, and Lifestyle Interventions

The effect of behavioral modifications in the management of prostate cancer patients cannot be underestimated. In obese patients, weight loss should be strongly recommended as a 5 kg/m^2^ increase in BMI among patients post-RP is associated with a 25% increased risk of BCR [69]. This association is sustained among patients who receive primary RT, with a 15% increased risk of BCR. Moreover, a low-carbohydrate diet in patients with BCR has been shown to significantly lengthen PSADT compared to control group (30 vs. 13 months; *p* = 0.007) when adjusted for baseline covariates [70].

Several supplements have been studied in the context of BCR prostate cancer. The strongest available evidence supports sulforaphane, a phytochemical which has shown promise as an anticancer agent among several malignancies. In a double-blinded, randomized, placebo-controlled trial of men with BCR post-RP, 60 mg of oral sulforaphane daily for 6 months significantly improved PSADT compared to placebo, 28.9 months vs. 15.5 months, respectively. Additionally, the rate of PSA rise > 20% at 6 months was significantly lower compared to placebo, 44.4% vs. 71.8%, respectively (*p* = 0.0163) [71]. Sulforaphane appears well-tolerated, with the main adverse effect being GI symptoms, specifically bloating.

In another placebo-controlled trial, Pomi-T (pomegranate, turmeric, green tea and broccoli sprout extract) showed a reduced rise in PSA compared to placebo, 8.8% vs. 80% in placebo [72]. Neither sulforaphane nor Pomi-T had a significant effect on serum testosterone levels.

Curcumin, a dietary polyphenol, may be useful as a complement to traditional management strategies. In a randomized, double-blind, placebo-controlled trial, curcumin was shown to inhibit prostate cancer growth and lower PSA progression rate compared to control (10% vs. 30%; *p* = 0.026) without affecting testosterone levels [73].

Other phytotherapeutics have also been evaluated including lycopene, soy isoflavones, and POMx (pomegranate extract), each showing limited effects on PSA dynamics [74–77]. High-quality studies on these supplements are lacking, with small study populations, and no long-term follow-up or data on effects on cancer-specific survival.

### ADT-Free Survival

Given the well-known and extensively described adverse effects of ADT, recent studies have begun exploring ADT-free survival as a novel clinical endpoint of interest [78–80]. As more emphasis is placed on patient QOL, it would be expected for this trend to continue generating novel data with hugely important clinical implications.

## Conclusion

Patients with BCR post-RP are a heterogeneous patient population. Risk stratification models integrating PSMA-PET imaging, genomics, and novel MMAI biomarkers are essential for personalized treatment approaches and are an area of ongoing research. Individualized treatment plans combining molecular profiling and targeted local therapies must be evaluated and implemented for BCR disease. Validated QOL tools must be developed and routinely evaluated to guide shared decision-making between physicians and patients. Contemporary literature supports selective TST in BCR prostate cancer patients without high-risk clinicopathologic features, but long term follow up is needed to further support widespread adoption.

## Of Outstanding Importance

- Spratt, D.E., et al., Artificial intelligence predictive model for hormone therapy use in prostate cancer. NEJM evidence, 2023. 2(8): p. EVIDoa2300023.
  - Demonstrates a validated AI-based predictive tool that may personalize ADT use and avoid overtreatment.
- Pollack, A., et al., The addition of androgen deprivation therapy and pelvic lymph node treatment to prostate bed salvage radiotherapy (NRG Oncology/RTOG 0534 SPPORT): an international, multicentre, randomised phase 3 trial. The Lancet, 2022. 399(10338): p. 1886-1901.
  - Practice-changing phase III evidence defining when pelvic nodal radiation and ADT should be combined in salvage settings.
- Deek, M.P., et al., Long-term outcomes and genetic predictors of response to metastasis-directed therapy versus observation in oligometastatic prostate cancer: analysis of STOMP and ORIOLE trials. Journal of Clinical Oncology, 2022. 40(29): p. 3377-3382.
  - This pooled long-term analysis of STOMP and ORIOLE shows metastasis-directed therapy can delay systemic therapy and prolong progression-free survival, providing strong clinical rationale for MDT as a testosterone-preserving option.
- Freedland, S.J., et al., Improved outcomes with enzalutamide in biochemically recurrent prostate cancer. New England Journal of Medicine, 2023. 389(16): p. 1453-1465.
  - Provides high-quality randomized evidence supporting enzalutamide monotherapy as an effective alternative to ADT with preserved testosterone levels.

## Data Availability

No datasets were generated or analysed during the current study.
